# Pancreatic steatosis and metabolic pancreatic disease: a new entity?

**DOI:** 10.1007/s11739-023-03364-y

**Published:** 2023-07-18

**Authors:** Federico Caldart, Nicolò de Pretis, Claudio Luchini, Rachele Ciccocioppo, Luca Frulloni

**Affiliations:** 1 Gastroenterology B Unit, University of Verona—Verona Hospital, Verona, Italy; 2 Department of Diagnostics and Public Health, Section of Pathology, ARC-Net Research Center, University and Hospital Trust of Verona, Verona, Italy

**Keywords:** pancreatic steatosis, fatty pancreas, pancreatic metabolic disease, pancreati fat accumulation

## Abstract

Overweight and obesity are some of the most important health challenges. Many diseases are related to these metabolic disorders, and, among them, the pancreatic fat accumulation, also called "pancreatic steatosis" or “nonalcoholic fatty pancreas”, seems to have an emerging role in different conditions. There are different method to evaluate the fat content in the pancreas, such as histology, different imaging techniques and endoscopic ultrasound, but there is no gold standard for the correct diagnosis and for the identification of “inter/intralobular” and “intra-acinar” pancreatic fat. However, the fat storage in the pancreas is linked to chronic inflammation and to several conditions, such as acute and chronic pancreatitis, type 2 diabetes mellitus and pancreatic cancer. In addition, pancreatic fat accumulation has also been demonstrated to play a role in surgical outcome after pancreatectomy, in particular for the development of postoperative pancreatic fistula. Different possible therapeutic approaches have been proposed, but there is still a lack of evidence. The aim of this review is to report the current evidence about the relationship between the obesity, the pancreatic fat accumulation and its potential role in pancreatic diseases.

## Introduction

Overweight and obesity are some of the most important health challenges worldwide, and their rates continue to grow in both adults and children, with a progressive increase in prevalence of more than fourfold in the last forty years (from 4 to 18% globally) [1]. According to the WHO, in 2016, more than 1.9 billion adults (39% aged 18 years and older) were overweight, and of these, over 650 million (18%) were obese (13%) [1]. Overweight (body mass index [BMI] between 25 and 30) and obesity (BMI > 30) are defined as abnormal or excessive fat accumulation, and they are also considered one side of the double burden of malnutrition [1].

Many diseases, including cardiovascular events (e.g., heart disease and stroke), diabetes, cancer (e.g., colon, endometrial, breast, ovarian, prostate, liver, gallbladder, and kidney neoplasms) and musculoskeletal disorders, are associated with obesity, with a wide range of serious complications, especially in children. Among obese subjects, increasing evidence shows the rise of a new entity due to fat accumulation in the pancreas, defined by various terms such as “pancreatic steatosis”[2], “pancreatic lipomatosis”[3], “fatty infiltration of the pancreas”[4] or “nonalcoholic fatty pancreas disease” (NAFPD)[5]. The prevalence of this condition is not yet precisely established, but different studies have reported a range between 16 and 35% [6, 7], depending on age and ethnicity. In fact, the volumes of total pancreas, pancreatic parenchyma, and fat increase linearly with age and with obesity [8], and different prevalence were reported between Asian and Western population (16% in Chinese population [6], 27% in a Western population [7], 35% in a cross-sectional study on south-east Asian cohort [9]).

In 2011, Smits et al. [10] proposed a definition of “fatty infiltration or nonalcoholic fatty pancreas” as the potentially reversible accumulation of fat in obese people, whereas the authors called “pancreatic fat replacement” the irreversible infiltration of fat after acinar cell death.

However, a widely accepted definition of this phenomenon has not yet been established.

### “Inter/intralobular” and “intra-acinar” pancreatic fat

Pancreatic fatty infiltration is a common pathologic finding, and it was first studied in the adult pancreas of unselected autopsies [11, 12]. In these studies, fatty replacement was mainly reported as patchy, and it was especially localized in the intralobular or “perilobular” area. At the macroscopic level, the adipose tissue involved less than one-quarter of the gland, whereas less than 10% of patients had no pancreatic fat infiltration [12].

Fatty replacement seems to also be correlated with age [11, 12], as this phenomenon is not usually observed in younger patients. Furthermore, the presence of fat in the pancreas can increase the weight of the gland, and it has been associated with the loss of parenchymal tissue, starting from the exocrine-acinar counterpart, justifying the use of the term “adipose atrophy of the pancreas” [12].

**Fig. 1:**
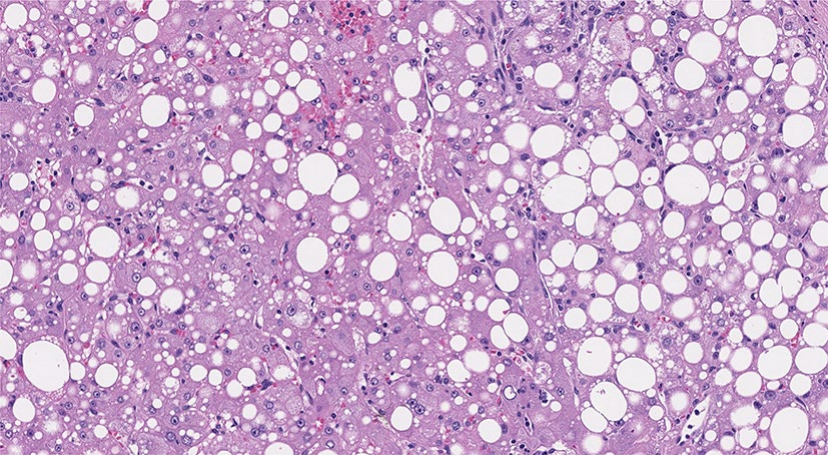
Hepatic “intercellular” macrovesicular steatosis (haematoxylin–eosin [HE] staining, 10 × original magnification, personal unpublished picture)

**Fig. 2:**
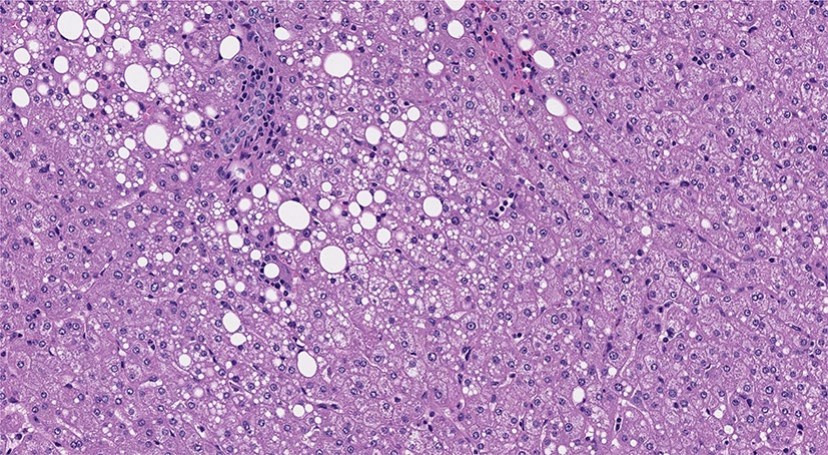
Hepatic “intracellular/microvesicular” steatosis: In this image, vacuolization of the cytoplasm of some hepatocytes is shown (haematoxylin–eosin [HE] staining, 10 × original magnification, personal unpublished picture)

**Fig. 3:**
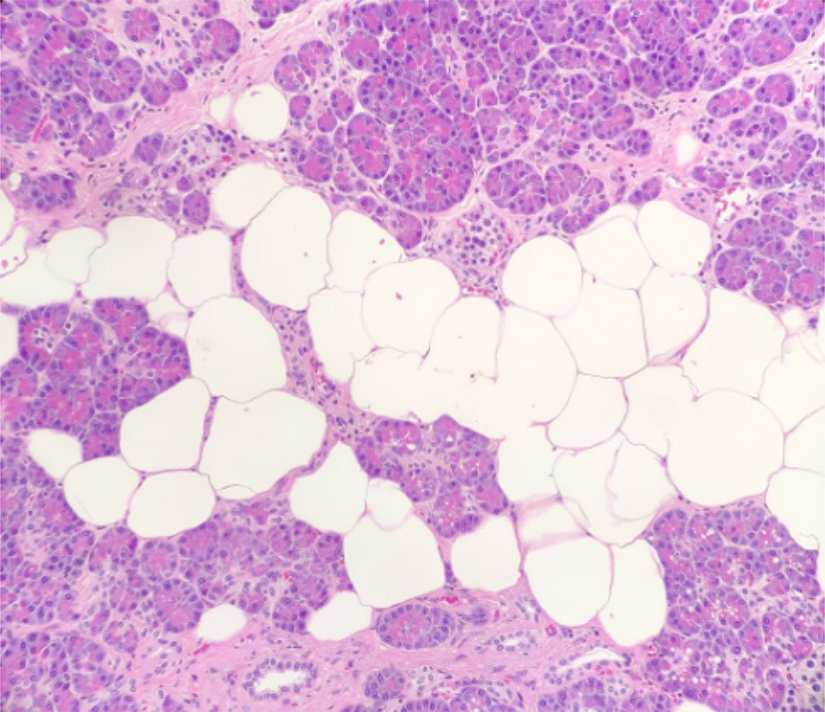
Pancreatic “intralobular” steatosis: This is a highly illustrative image showing the presence of marked adipose tissue infiltration into the pancreatic acinar parenchyma (haematoxylin–eosin [HE] staining, 10 × original magnification, personal unpublished data)

**Fig. 4:**
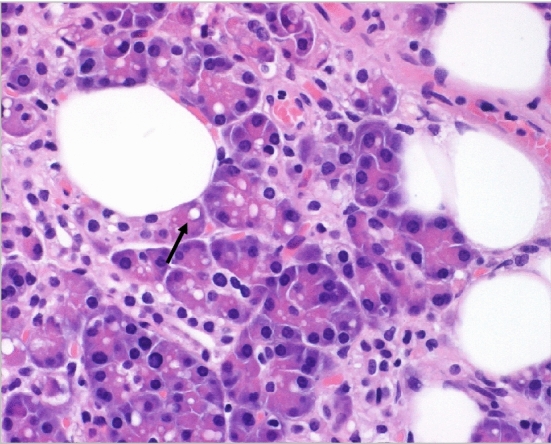
Particular “intra-acinar” fat distribution in the pancreas (haematoxylin–eosin [HE] staining, 10 × original magnification, personal unpublished data)

Therefore, pancreatic steatosis can be compared to hepatic steatosis, where the fatty infiltration is not only within hepatic cells but also around the hepatocytes (Figs. 1, 2). Some data suggest that the fatty infiltration might be intracellular (“intra-acinar”) even in the pancreas (Figs. 3, 4).

Yan et al. [13] demonstrated the presence of microvesicles in acinar cells in haematoxylin/eosin-stained sections of the pancreas in male Wistar rats fed a long-term high-fat diet (2% cholesterol, 10% lard, and 88% standard chow) compared with those fed a standard chow diet [13]. Specifically, other studies [14, 15] have also reported the accumulation and vacuolization of TG or other lipid metabolites in pancreatic cells, which was found among rats that were fed a high-fat diet.

Vacuolization has also been observed in humans (Fig. 4). In 1981, Noronha et al. reported the presence of large intra-acinar lipid droplets, probably related to the consumption of ethanol and its consequences on pancreatic lipid metabolism [16] In addition, in 1997, it was hypothesized that the accumulation of lipid droplets in acinar cells might interfere with the normal mechanisms of exocrine pancreatic secretion [17]. Later, Pinnick et al. [18] demonstrated, by using immunohistochemistry for perilipin, that fat is mainly stored in adipocytes between exocrine and islet cells in humans and in mice. In addition, they also found some adipose differentiation–related protein (ADFP)-positive and perilipin-negative intracellular vacuoles in pancreatic exocrine parenchyma in high-fat diet (HFD)-fed mice. Since ADFP is a marker of intracellular lipid droplets [19], this evidence confirmed that the vacuoles contain ectopic lipids, showing both “intra-acinar” pancreatic fat infiltration and hepatic steatosis in rats fed high-fat diets [13]. However, it is not clear if the main component of pancreatic fat infiltration is intralobular/perilobular or intracellular, although it can be assumed that the first component is much more represented.

Several implications of this concept need to be stressed. First, the fat content of the pancreas, evaluated by imaging investigation, might include both interlobular/intralobular and intra-acinar lipids. Furthermore, some pathological mechanisms of nonalcoholic fatty liver disease (NAFLD) or metabolic-associated fatty liver disease (MAFLD) might also be involved in the pancreas. Finally, the complications of pancreatic fat infiltration might be associated either with intralobular/perilobular or intra-acinar storage or both.

## Evaluation of the pancreatic fat content

There is no gold standard for the diagnosis of pancreatic fat, and different approaches have been proposed for the evaluation of fat storage in the pancreas.

Histology of pancreatic specimens allows for the detection of pancreatic infiltration of adipocytes and the localization of fat droplets within the acinar cells as reported above, but sampling of the pancreas is difficult due to its retroperitoneal location, and endoscopic access is generally preferred. However, tissue sampling is not indicated in the daily hospital setting in the absence of other indications, and there are no current histological correlations with clinical outcomes.

Imaging techniques are noninvasive tools for the detection and quantification of pancreatic fat, and they allow evaluation of the entire parenchyma. Currently, different methods have been proposed, including ultrasonography (US) [20], endoscopic ultrasound (EUS) [21], computerized tomography (CT) [20], proton magnetic resonance spectroscopy (H-MRS) [22] and MRI [23].

Transabdominal US is a noninvasive method with many advantages, such as wide availability or inexpensiveness, and pancreatic steatosis is usually diagnosed by comparing the pancreatic echogenicity with that of the kidney or spleen [24]. Nevertheless, US has several limitations, likely due to its operator dependence and to the features of the patients (e.g., intestinal air and obesity).

EUS has also been used to assess the fat content in the pancreas [21], but in addition to some limitations shared with US, it is a more complex procedure that requires anaesthesiologic support and more expertise by the endoscopist. A system with 4 grades was proposed by Sepe et al. [7] that compares pancreatic echogenicity with spleen echogenicity: grade I (hypo/isoechoic pancreas, clearly delineated pancreatic duct and parenchymal “salt and pepper” dots), grade II (hyperechoic pancreas, clearly delineated pancreatic duct and parenchymal “salt and pepper” dots), grade III (hyperechoic pancreas, pancreatic duct margins and parenchymal “salt and pepper” dots moderately obscured) and grade IV (severely hyperechoic pancreas, pancreatic duct margins and parenchymal “salt and pepper” dots severely obscured). According to this system, grades I and II represent normal pancreas, while grades III and IV correspond to fatty pancreas. However, to our knowledge, there is neither a standardized protocol for the quantification of pancreatic steatosis nor a study that investigated the sensitivity and specificity of these methods.

CT is another widely used and rapid method that is more sensitive and specific than US [25], with limitations such as ionizing radiation and the lack of a standardized imaging protocol. Nonenhanced CT is required, as fat density is altered by radiocontrast.

H-MRS is a further expensive technique for the assessment of fat accumulation in the pancreas, but its use is limited to research protocols due to its limited availability, and it requires high expertise.

Finally, MRI is the most commonly used method for the evaluation of the pancreas due to its high sensitivity and specificity for pancreatic alterations [26]. Specifically, MRI can be processed by the Dixon method to yield water- or fat-selection images, and proton density fat fraction identification is facilitated by the advanced multiecho Dixon technique [27] Despite these advantages, MRI has several limitations, such as high costs for research purposes, the need for expertise in pancreatic imaging and the wide availability of different imaging techniques.

## Pancreatic fat and acute and chronic inflammation: a complex relationship

The presence of fat in the pancreatic parenchyma is probably due to different but still unknown mechanisms (Table 1). Intracellular ectopic accumulation of lipid droplets and the infiltration of adipocytes, especially in the exocrine pancreas [17, 28], might lead to degenerative and inflammatory processes.

**Table 1:** Possible causes and mechanisms leading to pancreatic fat accumulation

Adiponectin and leptin on the adipocytes in the exocrine and endocrine pancreas [28, 29]	↑ Storage of triglycerides droplets
Accumulation of triglycerides in islet cells [30]	↓ Impairment in glucose sensing, glucokinase and insulin secretion
Introduction of exogenous long-chain fatty acids (palmitate, oleate and stearate) [31]	Modulation of insulin secretion
Increased plasma non esterified fatty acid (NEFA) levels by oral feeding [32]	↑ Glucose-stimulated insulin secretion
Increased monounsaturated fatty acids (MUFAs) by oral feeding [32]	↑ GLP-1 Insulin secretion
Chronically elevated levels of saturated long-chain fatty acids (SLCAs) [33]	Impairment of beta-cell survival
Free fatty acids (FFAs) and cytokines (hepatokine “fetuin-A” [34]	↑ Inflammation via TLR signalling (fetuin-A–IL-6–CXCL8–CCL2 cascade) “glucose blindness” of beta cells

Adipocytes are mostly localized in the exocrine pancreas and, to a lesser extent, in the endocrine part [29], and they store triglycerides in lipid droplets through the release of adiponectin and leptin [30] Obesity and the accumulation of fat in the pancreas are also related to the development of type 2 diabetes mellitus (T2DM) [31], with intracellular ectopic lipid storage in islet cells. The accumulation of triglycerides in islet endocrine cells has also been detected in mice and humans, and it was associated with an impairment in glucose sensing, glucokinase and insulin secretion [31]. In addition, the introduction of exogenous long-chain fatty acids, such as palmitate, oleate and stearate, might contribute to the modulation of insulin secretion [32], depending on the exposure time. Increased plasma nonesterified fatty acid (NEFA) levels by oral feeding augment glucose-stimulated insulin secretion, and monounsaturated fatty acids (MUFAs) also increase GLP-1 more than saturated fatty acids, leading to an exaggerated insulin concentration [33]. In addition, chronically elevated levels of saturated long-chain fatty acids (SLCAs) impair beta-cell survival [34].

Increased levels of adipose tissue in the pancreas are associated with low-grade chronic inflammation since adipocytes produce proinflammatory cytokines, chemokines and chemoattractants [32]. Free fatty acids (FFAs) and cytokines, such as the hepatokine “fetuin-A” [35], increase local inflammation via TLR-dependent signalling. Specifically, human islets have been shown [36] to contain more CD68 + macrophages and monocytes if they are in close proximity to pancreatic adipocytes compared with those far from them, suggesting a role in an inflammatory TLR4-dependent pathway (via the fetuin-A–IL-6–CXCL8–CCL2 cascade), causing “glucose blindness” of beta cells.

Therefore, these physio-pathogenetic mechanisms depend on different factors, not only on the direct release of free fatty acids during lipolysis, but also on various metabolites, cytokines, chemokines and adipokines, that influence the function of islet cells in a way that is not completely understood.

## Conditions associated with pancreatic fat accumulation

Pancreatic fat accumulation has been investigated in different conditions, such as obesity, age, BMI, metabolic syndrome, nonalcoholic fatty liver disease (NAFLD – better defined as “metabolic associated fatty liver diseases” or “MAFLD”) and alcohol consumption.

Several studies [7, 24, 37] conducted in heterogenic ethnic groups have found an association between pancreatic steatosis and metabolic syndrome (defined according to NCEP-ATP III criteria as abdominal obesity, hypertension, dyslipidaemia and insulin resistance or overt T2DM).

Increased BMI and obesity, especially visceral adipose tissue, have been demonstrated to be linked to fat accumulation in the pancreas, independent of sex and age [8, 38–40].

Non-alcoholic fatty liver disease (NAFLD) is a well-known condition clearly related to obesity, metabolic syndrome and T2DM, and it has been investigated to find possible associations with pancreatic steatosis [39, 41, 42]. Controversial results have been demonstrated, and this association might be related to other variables (e.g., obesity itself and increased visceral adipose tissue). Taylor et al. [43] (DIRECT trial) first reported that in diabetic people, increased mobilisation of triglycerides from the liver could lead to pancreatic fat accumulation, resulting in inadequate secretion of insulin with high levels of serum glucose. Using magnetic resonance spectroscopy (MRS) in 36 Dutch volunteers with a body mass index (BMI) ranging between 20.0 and 42.9 kg/m2, a significant correlation between the liver and pancreatic fat content (r = 0.43, p < 0.01) has been shown [44]. Moreover, an increased prevalence of pancreatic steatosis in patients with NAFLD was observed in a study from California (USA) [45] However, the severity or grade of activity of NAFLD has not been demonstrated to be associated with progressive fat infiltration in the pancreas [42].

Furthermore, ageing is another factor that is consistently linked to pancreatic steatosis[8, 28, 46]: in a large cohort of children and adults, both nondiabetic and subjects with T2DM, Saisho et al. [8] found that from age 20–60 years, pancreas size reached a plateau, which was increased with obesity in terms of total, parenchymal and fat proportions, but after 60 years, a higher fat content has been reported with a decreased volume of pancreatic parenchyma.

Alcohol is a well-known risk factor for acute pancreatitis [47, 48] and for hepatic steatosis: al-Haddad et al. [21] showed that hepatic steatosis, alcohol intake (> 14 g/week) and increased BMI, by multivariate logistic regression, are predictors of hyperechogenic pancreas, compared to liver or spleen, on EUS imaging.

## Pancreatic fat leads to pancreatic diseases

The presence of fat in the pancreatic parenchyma causes chronic inflammation, which might lead to fibrotic replacement of the acinar cells and probably to neoplastic degeneration, but this matter remains unknown.

### Acute pancreatitis

Obesity and overweight were usually part of metabolic syndrome, defined as a cluster of common abnormalities, including insulin resistance, impaired glucose tolerance, abdominal obesity, reduced high-density lipoprotein (HDL)-cholesterol levels, elevated triglycerides, and hypertension according to NCEP-ATP-III criteria [49].

In this context, a role of the triglycerides is also known in the physiopathology of a type of acute pancreatitis: in fact, hypertrigyceridemic (HTG) pancreatitis typically occurs in patients with an underlying dyslipidaemia (such as type I, IV or V), where HTG, due to an excess of free fatty acids, and elevated chylomicrons are thought to increase plasma viscosity, inducing ischemia in pancreatic tissue and trigger organ inflammation [50].

In addition, obesity and lipolytic unsaturated fatty acid may contribute to increasing and worsening acute pancreatitis [51]. During the acute episode, the leaked lipases hydrolyse the adipocytes and this can generate a pancreatic necrosis, enriched in the unsaturated fatty acids (UFAs), in particular in oleic (C18: 1) and linoleic acid (C18: 2) [52]. The UFAs have been shown to have a role as lipid mediators in the severe acute pancreatitis. As polar molecules, they are bound by calcium, resulting in their saponification and inactivation in fat necrosis [51] and also in the hypocalcemia. Inflammatory mediators, such as tumor necrosis factor (TNF-a), CXC ligand 1 (CXCL1), and CXCL2, are increased by the remaining unbuffered non-esterified UFAs, that inhibit mitochondrial complexes I and V, reducing ATP levels: this mechanism contributes to the pancreatic necrosis, thus worsening acute pancreatitis [51, 53].

However, we hypothesize that the intracinar fat accumulation might damage the exocytosis of the pancreatic enzymes, leading to an uncontrolled release of them and finally to acute pancreatitis. We speculate that the pancreatic steatosis, especially into the acinar cells, might have a key role in the development of acute episode of pancreatitis and in the worsening of it, due to the alterations of the normal intracellular processes and of the pancreatic omeostasis.

### Chronic pancreatitis

In a retrospective study [54], Tyrkes et al. showed that patients with chronic pancreatitis (CP), as well as those with T2DM, have higher visceral fat, demonstrating that increased visceral adipose tissue has a moderate correlation with the pancreatic fat fraction [38, 39]. In another prospective study [55] from 2008 to 2014, Fujii et al. found that fat accumulation could be a risk factor for developing subclinical chronic pancreatitis (adjusted OR 3.96, 95% CI 2.04–7.66) in ninety-nine patients with CP who underwent a medical check-up for pancreatic steatosis.

### Type 2 diabetes mellitus

The relationship between pancreatic fat and T2DM has been deeply investigated only in the last few years, showing a close association between these two conditions in most studies [56–58], if not in all [59, 60]. In a large cohort of nondiabetic lean individuals followed for approximately 6 years, Yamazaki et al. [61] found that T2D might develop in individuals with fatty pancreas. A potential role of fatty pancreas and its correlation to the pathogenesis of T2DM has been further investigated by several studies, which tried to demonstrate a link among insulin resistance, compromised pancreatic β-cell function and fat accumulation in the pancreas. Using proton magnetic resonance spectroscopy (H-MRS), Begovatz et al. [62] showed that there was no relationship between interlobular/intralobular fat storage and abnormal endocrine function, as have many other emerging studies [23, 62, 63]. No clarifying evidence supports the hypothesis of insulin secretion impairment related to fatty parenchymal replacement, since different factors, such as the type of population (e.g., lean individuals vs. obese people), imaging technique or ethnicity, might influence the results reported below. Although lipogenesis and adipocyte differentiation are stimulated by insulin, the crosstalk among pancreatic islets, serum blood glycaemia and counterregulatory molecules (e.g., glucagon, adrenaline and noradrenaline) may be modified by the pancreatic adipose fraction. Emerging evidence [64] in mice showed an active impairment in insulin secretion by fatty acids released by pancreatic adipocytes. On the other hand, hereditary factors have also been investigated [29], and individuals with high genetic risk seem to have a negative association between pancreatic fat and insulin secretion, suggesting that pancreatic steatosis impairs only beta-cell function, especially in genetically determined insulin resistance. However, not only insulin secretion but also insulin resistance could be associated with pancreatic fat: in two studies [20, 57], fatty pancreas was associated with higher insulin resistance (measured by HOMA-IR, associated with visceral fat area, triglycerides, and elevation of ALT) [20], especially in male T2DM subjects with a shorter duration of diabetes [57]. However, T2DM and prediabetes are complex and heterogeneous conditions with different subphenotypes [65] based on glycaemic and lipid profiles, body fat distribution and genetic risk.

### Pancreatic cancer

Obesity is a key risk factor for the development of different types of pancreatic cancers, particularly pancreatic ductal adenocarcinoma (PDAC) [4, 66–69]. Of note, the accumulation of adipose tissue within the pancreas can contribute to pancreatic oncogenesis from existing nonalcoholic fatty pancreas disease [70]. A recent investigation involving surgically resected pancreatic specimens clearly showed a strong association between adipose tissue infiltration in the pancreatic parenchyma and PDAC, as confirmed by multivariable analysis [66]. Furthermore, the well-established microscopic PDAC precursor known as PanIN (pancreatic intraepithelial neoplasia) also showed a strong association with fatty infiltration, suggesting a potential role of pancreatic steatosis in the early phases of PDAC oncogenesis [4, 67]. Since the presence of pancreatic steatosis has been associated with clinicopathologic variables of aggressive disease, such as an increased metastatic lymph node ratio, this pathological condition may also play a role in the late phases of PDAC, including tumour spread and nodal dissemination [2]. When histologically specified, fat accumulation within the pancreas shows an inter/intralobular pattern, with a substitution of exocrine/endocrine parenchyma with adipose tissue [71] Furthermore, as observed in the liver, the presence of intra-acinar steatosis may also modify the intracellular metabolism of fatty acids, with the release of related molecules preliminarily described in pancreatic cancer [72]

However, the dilemma remains open: supporting data have shown that the accumulation of fat in the pancreatic parenchyma could impair insulin secretion, but whether metabolic syndrome and visceral fat could interfere with this process or contribute to the development of T2DM and other pancreatic diseases (e.g., chronic pancreatitis and cancer) is still debated.

## Pancreatic fat accumulation and pancreatic surgical outcome

Pancreatic fat accumulation has also been demonstrated to play a role in surgical outcome after pancreatectomy [73–75]. The most frequent complications reported after pancreatectomy are delayed gastric emptying, postoperative haemorrhage, and postoperative pancreatic fistula (POPF). Among these, POPF is the most threatening complication, causing intra-abdominal infections, severe sepsis, and massive bleeding [76], with a prolonged hospital stay and high health care costs.

In a study of forty patients, Mathur et al.[75] showed that pancreatic fistula was more likely to occur in patients with a high pancreatic fat score based on the intralobular and interlobular fat content on histology. Similar results were reported by Gaujoux et al. [73] in a study of 100 consecutive patients who underwent pancreatoduodenectomy (PD) that considered different fat infiltration within and between the lobules, which was quantified with a histological score ranging from 0 to 4. More recently, a Japanese group [74] confirmed this evidence, demonstrating that patients with a soft pancreas, a thick parenchyma, a small main pancreatic duct, and fatty infiltration were strongly associated with clinically relevant POPF after PD.

CT measurement of the fatty pancreatic content has been investigated as a predictive tool for the development of POPF, with conflicting results between PD and distal pancreatectomy. In fact, Maeda et al. [76] evaluated the pancreas-visceral fat CT value ratio and serrated pancreatic contour using preoperative CT, reporting that they were not risk factors for POPF after distal pancreatectomy. In contrast, another Japanese group [77] demonstrated on 150 consecutive patients who underwent curative pancreatectomy that POPF was significantly associated with a high ﻿ratio of pancreatic fat (RPF), determined by the pancreatic fat volume/pancreatic volume on CT scan (using the Hounsfield unit thresholds of − 200 to − 50).

In addition, Angrisani et al. [78] have recently demonstrated that the assessment of preoperative fat mass by bioimpedance vector analysis (BIVA) can improve the accuracy of the “fistula risk score” in predicting clinically relevant postoperative pancreatic fistula (CR-POPF) following PD, since a high preoperative fat content measured by BIVA was found in patients who developed CR-POPF.

In conclusion, fatty infiltration of the pancreas has relevant consequences not only for the development of pancreatic diseases but also for important postoperative complications that can modify the surgical outcome.

## Possible therapeutic approaches

In this multifactorial scenario, the therapeutic target is to reduce pancreatic fat with different approaches.

### DIET

Diet plays an important role in metabolic syndrome, obesity and NAFLD, and it seems to have a similar effect on pancreatic fat. In animal models (mice), a high-fat diet led to islet degeneration, interlobular adipocyte accumulation and vacuolization in pancreatic tissue, suggesting possible glucolipotoxic effects on the pancreas, which depend on the ratio of saturated to unsaturated fatty acids [79]. In contrast, treatment with the fermented food-rich sodium butyrate improved insulin secretion and lowered lipid accumulation [80].

### Weight loss

Weight loss after gastric bypass surgery and restricted energy intake have been reported to lower the content of intrapancreatic triglycerides in patients with T2DM [43]. Generic lifestyle advice, such as sufficient exercise, a balanced diet, and healthy weight, might be effective in reducing pancreatic fat, but there is a lack of evidence. Limited data from studies on mice (NZO mice) [64] have shown that intermittent fasting not only improved glucose homeostasis and insulin resistance but also lowered fat accumulation in both the pancreas and the liver.

### GLP-1 receptor agonists

To our knowledge, only GLP-1 receptor agonists have been tested for reducing fat storage in the pancreas. A prospective randomized trial [81] was conducted in 44 obese subjects with T2DM uncontrolled with oral antidiabetic drugs in which they randomly received exenatide or reference treatment, and the results showed no statistically significant difference in the pancreatic triglyceride content evaluated with 3T magnetic resonance imaging (MRI) and proton magnetic resonance spectroscopy.

Liraglutide has also been investigated in a single-centre randomized double-blind trial [82] in seventy-one patients with long-standing T2DM that measured the changes in insulin and glucagon secretion and performed magnetic resonance for the evaluation of subcutaneous, visceral and ectopic fat in the liver and pancreas; this study showed no improvement in the pancreatic fat content. In a 24-week open-label RCT [83] in individuals with T2DM and NAFLD, Kuchay et al. evaluated the effect of dulaglutide in modifying the content of the fat fraction by MRI-derived proton density quantification, but they did not find a difference in the pancreatic fat content between the dulaglutide group and the control group.

### Pioglitazone

Another possible target is peroxisome proliferator-activated receptor-γ (PPAR-γ), which regulates gene transcription, and its agonists are antidiabetic agents with pleiotropic metabolic effects [32]. Pioglitazone [84] has been shown to reduce fasting triglycerides and FFA levels, with an improvement in the insulin sensitivity of lipolysis, which might be useful in decreasing pancreatic fat accumulation.

However, further studies are necessary to establish a valid therapy for reducing fat storage in the pancreas, with an effective clinical outcome.

## Conclusions

The presence of pancreatic adipocytes and their possible role in the development of pancreatic diseases, such as T2DM, chronic pancreatitis, and cancer, must be further investigated.

Growing evidence suggests that pancreatic fat is a favourable setting for a pathologic microenvironment, especially with an impairment in beta-cell function and insulin secretion. How it could also damage the exocrine compartment remains unclear. We speculate that the identification of different levels of pancreatic fat might be useful in the future to stratify those at risk of T2DM and, perhaps, chronic pancreatitis. Obesity is a disease of the “not too distant” future, and from this perspective, improving lifestyle is mandatory to prevent cardiovascular, liver and especially pancreatic disease.

## Data Availability

The data used to support the findings of this study are included within the article.
